# The potential utility of the SAGIT instrument in the clinical assessment of patients with acromegaly, a large single-centre study

**DOI:** 10.1038/s41598-023-29957-3

**Published:** 2023-02-25

**Authors:** Nadia Sawicka-Gutaj, Paulina Ziółkowska, Aleksandra Biczysko, Abikasinee Erampamoorthy, Katarzyna Ziemnicka, Marek Ruchała

**Affiliations:** Department of Endocrinology, Metabolism and Internal Medicine, Poznan University of Medical Sciences, Poznan, Poland

**Keywords:** Endocrinology, Pituitary diseases

## Abstract

SAGIT is an instrument created for the clinical assessment of acromegaly. Our objective was to test the usefulness of this tool in assessing disease activity of acromegalic patients in a single centre of Poznan, Poland using a retrospective study. Medical records of patients with acromegaly hospitalised at the Department of Endocrinology, Metabolism and Internal Medicine of Poznan University of Medical Sciences in Poland between January 2015 and December 2020 were analysed. SAGIT scores were assessed according to each patient's clinical and biochemical data. The results show that SAGIT scores were higher in treatment-naïve patients and the lowest in controlled patients. There were positive correlations between SAGIT scores and concentrations of calcium, phosphorus, HbA1C levels, and tumour invasiveness at the time of diagnosis. However, parameters such as age, vitamin D concentration, and time from diagnosis showed an inverse relationship with the SAGIT score. In ROC curve analysis, SAGIT scores of 5 or less discriminated controlled patients from uncontrolled (p < 0.0001, sensitivity 76.7%, specificity 78.5%, AUC 0.867). Also, SAGIT higher than 6 indicated for treatment start or escalation (p < 0.0001, sensitivity 80.88%, specificity 77.59%, AUC 0.866). Lack of signs and symptoms (S = 0) could not discriminate between controlled and uncontrolled disease, but predicted therapy maintenance (p < 0.0004, sensitivity 59.5%, specificity 58.2%, AUC 0.604). In conclusion, The SAGIT instrument is easy to use even when completed in the retrospective medical record review. It can be useful for distinguishing clinical stages of acromegaly and in decision-making.

## Introduction

Acromegaly is a chronic disease caused by growth hormone (GH)-secreting pituitary adenomas^1^, leading to increased levels of insulin-like growth factor (IGF-1). Hence, some profound somatic changes are observed, such as enlarged hands and feet, thickened skin, mandible growth, joint tenderness, and enlarged facial features involving the facial bones, lips, nose, and tongue^2,3^. Other symptoms of acromegaly include headaches, excessive sweating, snoring or apnea, changes in voice timbre, weight gain, and sexual dysfunction^2,3^. These signs and symptoms significantly reduce the quality of life of patients suffering from acromegaly^4,5^. In addition, acromegaly leads to the development of many diseases, particularly affecting the cardiovascular system (hypertension, heart failure), and metabolism (diabetes), and increasing the risk of various cancers (thyroid cancer, colorectal cancer)^6–10^. These associated comorbidities in turn, also lower the quality of life and shorten life expectancy^11,12^.

The goals of acromegaly therapy, such as neurosurgical treatment and somatostatin analogues are mainly to normalise GH and IGF-1 levels, lower symptoms, reduce the risk of developing comorbidities, and prolong survival^13,14^. Therefore, it is essential to balance the delay of the intensification of therapy with the overtreatment of the disease. It is also crucial to consider the patient's well-being when making therapeutic decisions along with monitoring the intensity of symptoms typical for acromegaly^15^. Therefore, the search for a tool began that would be of decisive importance and would also consider both clinical and biochemical parameters in the care of acromegalic patients.

The SAGIT instrument was created to help endocrinologists care for patients with acromegaly in everyday clinical practice by providing crucial information about the disease activity and severity and assisting in the therapeutic decision-making process^16,17^. SAGIT is an acronym which reflects each part of the tool: signs and symptoms (S), associated comorbidities (A), GH levels (G), IGF-1 levels (I) and tumour profile (T)^16,17^. Recently, SAGIT was recognised as a sensitive tool, helpful in the management of acromegaly^18^. Nevertheless, studies have shown that acromegalic clinical features and complications differ between countries and cultures^19^. Therefore, investigating this tool in a Poland cohort can add more information in this field.

In this study, we aimed to test the usefulness of SAGIT in assessing disease activity and establishing therapeutic decisions in acromegalic patients of an endocrine centre in Poznan, Poland. For this purpose, a retrospective single-centre study based on patients' medical history was conducted.

## Patients and methods

### Study design

Medical charts of adult patients with acromegaly hospitalised at the Department of Endocrinology, Metabolism and Internal Medicine of Poznan University of Medical Sciences in Poland between January 2015 and December 2020 were retrospectively reviewed. Clinical and biochemical data were collected. SAGIT instrument was completed using patients' medical records. Patients were divided into three categories: stable/controlled; active/uncontrolled and treatment-naïve. Also, treatment decisions were recorded as continuing current therapy with no change/no treatment initiation; intensifying current therapy/initiating a treatment; or reducing the current treatment. Acromegaly was diagnosed according to the current guidelines^20^.

### Ethics approval

The Bioethical Committee of Poznan University of Medical Sciences approved this study and waived the requirement for informed consent due to the retrospective nature of the study (Decision No 633/22). All methods were performed in accordance with the relevant guidelines and regulations^21^.

### Clinical and laboratory assessment

At admission, every patient had a medical interview and underwent a physical examination. Blood samples of all patients were taken after overnight fasting. IGF-1 and GH were measured. According to guidelines, nadir GH in the oral glucose tolerance test was measured in all non-diabetic patients. In patients with diabetes mellitus and in those, who had already been treated with somatostatin analogues, random GH was calculated as an arithmetic mean of five measurements of blood samples obtained every 30 min. MRI scan of the pituitary gland was also performed routinely in all patients with acromegaly unless there were no contraindications. Treatment naïve acromegaly was diagnosed when all of the following criteria were fulfilled:

- IGF-1 elevated above the age-adjusted upper norm limit
- Lack of suppression of GH below 1 ng/mL in 75 g oral glucose tolerance test (patients without diabetes) or random GH levels above 2.5 ng/mL (patients with diabetes)
- A pituitary gland tumour visualised in magnetic resonance imaging (MRI) or computed tomography (CT) (in patients with contraindications for MRI).

Previously treated patients who achieved age-normalisation of IGF-1 and normalisation of GH (GH < 1 ng/mL in 75 g OGTT or random GH < 2.5 ng/mL in diabetic patients) were classified as a stable/controlled group. Acromegaly was considered active/uncontrolled (non-remission group) when IGF-1 and GH were elevated in patients who had already been treated. Patients who could not be classified according to the above-mentioned criteria were excluded from the analysis. Tumour invasiveness was defined as the infiltration of surrounding tissues.

### SAGIT instrument

The SAGIT instrument was created to assess the clinical disease activity of acromegaly and assist in making therapeutic decisions. SAGIT is an acronym reflecting key components of acromegaly: signs and symptoms (S), associated comorbidities (A), GH levels (G), IGF-1 levels (I) and tumour features (T)^16,17^. Each of the above units is scored by the clinician: S(0–4), A(0–6), G(0–4), I(0–3), and T(0–5). The higher the score in each category and the total sum of points, the greater the advancement of a given factor and overall disease activity^16–18^. Therefore, this tool assesses clinical and biochemical factors, ensuring a comprehensive estimation of the patient's condition. The SAGIT has been recently validated by performing an international multicentre, non-interventional validation study^18^.

### Statistical analyses

MedCalc® Statistical Software version 20.015 (MedCalc Software Ltd, Ostend, Belgium; https://www.medcalc.org; 2021) was used to perform statistical analysis. Normality was assessed by the D'Agostino-Pearson test. Comparisons between two and three groups were completed with the Mann Whitney and Kruskal Wallis tests, respectively. The Spearman Rank Correlation test was used to find an association between analysed parameters. To determine SAGIT utility to reflect the clinical status of acromegaly, receiver operating characteristics (ROC) curves were calculated. A P-value less than 0.05 was considered statistically significant.

### Ethics approval

The Bioethical Committee of Poznan University of Medical Sciences approved this study and waived the requirement for informed consent due to the retrospective nature of the study (Decision No 633/22)^21^.

## Results

### Patients and patient-admissions

Three patients were excluded as they did not fulfil all criteria of remission/non-remission groups. Finally, 316 hospitalisations of 175 patients (53 patients were hospitalised twice, 15 were hospitalised three times, ten patients were hospitalised four times, and seven patients were hospitalised five times) were included for analysis. Two hundred-two admissions were of female patients (63.9%). The median age of patient admissions was 58 years (IQR 45–64 years), and the median time of disease duration was 48 months (IQR 9–132 months). Median BMI was 28.3 kg/m^2^ (IQR 25.4–32.5 kg/m^2^). There were 55 treatment-naïve patients. One hundred forty-five patient admissions of previously treated patients (transsphenoidal surgery/current pharmacotherapy/radiotherapy) had active acromegaly: 35 were treated with octreotide, 70 with lanreotide, and three with pasireotide. Other clinical and biochemical data are presented in Table 1.

**Table 1:** Clinical and biochemical data of three study groups.

Characteristic	Naïve N = 55	Non-remission group N = 145 patient-admissions	Stable/controlled group N = 116 patient-admissions	p
Gender (M male; F female)	M 29 F 26	M 50 F 95	M 35 F 81	**0.0141**
Age (years) Me (IQR)	(n = 55) 54*# (39–62)	(n = 145) 56*(45–65.25)	(n = 116) 59#(47–64.5)	**0.041124**
BMI (kg/m^2^) Me (IQR)	(n = 51) 28 (24.7–30.7)	(n = 134) 28.1 (25.5–32.6)	(n = 106) 29 (25.7–33.6)	0.5583
Duration time from diagnosis (months) Me (IQR)	**NA**	**(n = 140)** **48 (21–99.5)**	**(n = 132)** **132 (48–187)**	** < 0.0001**
Tumour size (mm) Me (IQR)	**(n = 55)** 11 (9–14)*	**(n = 137)** **11 (0–17.25)#**	**(n = 113)** 0 (0–8.3)*#	** < 0.000001**
SAGIT Me (IQR)	**(n = 55)** 11 (9–14)*	**(n = 145)** 8 (5–11)*	**(n = 116)** 3 (3–5)*	** < 0.000001**
IGF-1 [ng/mL] Me (IQR)	**(n = 55)** 702 (544.25–921)*	**(n = 145)** 342 (219.5–568.5)*	**(n = 116)** 167 (116–214)*	** < 0.000001**
nadir GH in OGTT	**(n = 35)** **6.91 (3.05–18.3)**	**(n = 77)** **2.35 (1.52–4.33)**	**(n = 84)** **0.62 (0.28–0.88)**	**p < 0.000001**
GH random	**(n = 20)** **9.71 (4.49–17.02)**	**(n = 68)** **4.24 (1.96–8.8)**	**(n = 32)** **0.724 (0.44–1.34)**	**p < 0.000001**

Significant values are in [bold].

Me, median; IQR, interquartile range; BMI, body mass index; OGTT, oral glucose tolerance test; IGF-1, insulin-like growth factor; GH, growth hormone.

Note: Data marked with the same markers differ significantly.

### SAGIT

**Figure 1:**
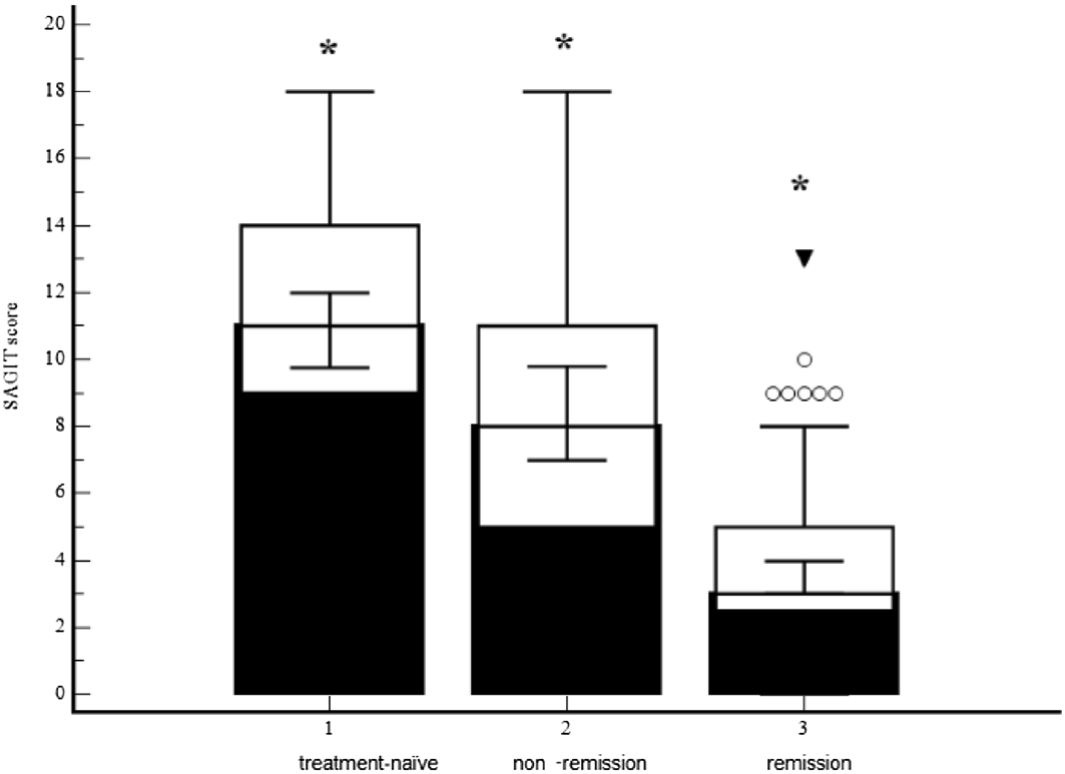
SAGIT score comparison between three study groups.

The highest median SAGIT global score was in treatment-naïve patients, and the lowest was in controlled patients (p < 0.000001, Fig. 1). There was an inverse correlation between patients' age and SAGIT score (p = 0.0397; r = − 0.122). SAGIT global score correlated with tumour invasiveness at the diagnosis (p = 0.0009; r = 0.294).

### SAGIT components

"S" component (signs and symptoms) was higher in de novo acromegalic patients, while there was no difference between active and non-active patients. "A" component (associated comorbidities) was higher in the non-remission group. Comparisons of all SAGIT components are presented in Table 2.

**Table 2:** Comparison between SAGIT components of three study groups.

	Naïve N = 55	Non-remission group N = 145 patient-admissions	Stable/controlled group N = 116 patient-admissions	p
S	1 (0–2)*#	0 (0–1)*	0 (0–1)#	0.003176
A	2 (1–2)*	2 (1–3)*#	2 (1–3)#	0.01
G	4 (2–4)*	2 (1.75–3)*	0 (0–1)*	< 0.000001
I	3 (2–3)*	1 (0–3)*	0 (0–0)*	< 0.000001
T	2 (1–4)*	2 (0–4)*	0 (0–1)*	< 0.000001

Note: Data marked with the same markers differ significantly.

### Association between SAGIT and laboratory parameters

Higher SAGIT was associated with HbA1C and fasting glucose levels (p < 0.0001; r = 0.394 and p < 0.0001; r = 0.368, respectively). Also, SAGIT positively correlated with calcium and phosphorus concentrations (p < 0.0001; r = 0.335 and p < 0.0001; r = 0.476, respectively), while there was an inverse association with vitamin D (p = 0.0156; r = − 0.192) and time from diagnosis (p < 0.0001; r = − 0.410).

"S" component correlated with IGF-1 concentrations (p < 0.0001, r = 0.263), glucose levels (p = 0.005, r = 0.158), time from diagnosis (p = 0.0039, r = − 0.165), and tumor size (p = 0.0001, r = 0.226).

### SAGIT as a predictor of further therapy

In ROC curve analysis, SAGIT scores of 5 or less discriminated controlled patients from uncontrolled (p < 0.0001, sensitivity 76.7%, specificity 78.5%, AUC 0.867). Also, SAGIT higher than 6 indicated for treatment start or escalation (p < 0.0001, sensitivity 80.88%, specificity 77.59%, AUC 0.866). Lack of signs and symptoms (S = 0) could not discriminate between controlled and uncontrolled disease, but predicted therapy maintenance (p < 0.0004, sensitivity 59.5%, specificity 58.2%, AUC 0.604). Also, S higher than 0 indicated for treatment initiation/intensification (p < 0.0001, sensitivity 63.24%, specificity 60.92%, AUC 0.644).

## Discussion

In this study, we investigated the usefulness of the SAGIT instrument in assessing disease activity and making therapeutic decisions during the management of patients with acromegaly in a single centre in Poznan, Poland. We found that the SAGIT score differed between disease activity groups and was the highest in naïve treatment but lowest in the stable/controlled group. These findings agree with the results shown in the SAGIT validation study^18^. Therefore, this supports the usefulness and credibility of this tool in the comprehensive assessment of disease activity in everyday clinical practice. Furthermore, we observed that the SAGIT-S component does not differ between controlled and uncontrolled disease. This conclusion raises a crucial issue which suggests that the normalisation of the biochemical parameters of disease activity does not equal a clinical cure for the disease. Many morphological changes in the body during acromegaly occur irreversibly, implying that these symptoms change in a continuous manner of greater or lesser intensity. This should be borne in mind when making therapeutic decisions. SAGIT, which contains both clinical and biochemical components, will be very helpful in this potential issue^18^.

It is essential to mention the correlation of the SAGIT global score with specific biochemical parameters. There was also a strong association between SAGIT scores and fasting glucose and HbA1C levels. The effect of acromegaly and GH on glucose regulation is extensively studied. Insulin resistance and the development of diabetes mellitus as a complication have been established in patients with acromegaly^22,23^. It has been recently reported, that 95% of patients with acromegaly suffer from comorbidities^24^. Therefore, the presence of associated comorbidities (A) in SAGIT is an advantage in assessing patients with acromegaly. We also observed a positive correlation between the SAGIT global score and the serum phosphorus concentration. It is well known that hyperphosphatemia is observed in patients with acromegaly. However, research on this issue is still ongoing, including in the context of disease activity^25,26^. Xie et al., concluded in their study that the level of phosphorus reflects the disease status as a product of metabolism. Moreover, it can help monitor the disease with divergent GH and IGF1 values^26^. Another positive correlation that we noticed is between the global score of SAGIT and the level of calcium. Mild hypercalcemia in patients with acromegaly is common and primarily parathyroid hormone-dependent which occurs as a result of concurrent parathyroid hyperplasia in patients with MEN-1. However, overt hypercalcemia in patients with acromegaly is very rare and is associated with parathyroid hormone-independent hypercalcemia^27^. Few cases have been reported, with authors concluding that the mechanism behind this is related to an increased level of 1,25 dihydroxy vitamin D^28,29^. Shi et al., point out that activation of 1-alpha hydroxylase by increased levels of IGF-1 in acromegaly could potentially be the cause of this phenomenon. The combination of absorption of calcium from the gut and kidney with increased bone turnover contributes to this. They also pointed out that hypercalcemia in these patients is reversible as remission of acromegaly is achieved^30^. Conversely, our study showed an inverse relationship between vitamin D levels and SAGIT scores. Researchers have also explored the potential vitamin D deficiency in acromegalic patients^31^. Therefore, the relationship between acromegaly and vitamin D regulation remains a complex topic and needs further research.

We also found higher SAGIT scores among younger patients. The impact of age on endocrine parameters of acromegaly was studied by Colao et al., Their results show that IGF-1 levels, GH levels, and nadir GH after glucose load are inversely related to age. In addition, older patients had smaller adenomas than younger patients at the time of diagnosis^32^. Also, a study by Park et al.^33^ showed that younger patients with acromegaly tend to have more aggressive adenomas and biochemically hyperactive disease. Recently, a study evaluating the gender and age differences among acromegalic patients demonstrated that hyperprolactinemia, hypogonadism and macroadenomas are more frequent in younger patients^34^. However, it is also important to note that this finding could be attributed to the age differences of groups, with the treatment-naive group being younger than the disease-controlled group. Although research on this correlation is limited, age appears to affect acromegaly's clinical and biochemical parameters. Thus, the SAGIT system again demonstrates its excellency in assessing various aspects of the disease.

The strengths of our analysis are, among others, a large research group and the analysis of SAGIT correlation with biochemical parameters. The study's retrospective nature is both positive- it proves that SAGIT can be determined based on medical records, and negative, as retrospective design leads to an information bias. It further illustrates the additional benefit of the SAGIT tool in that it can be assessed with ease without the use of any other third-party tools. The single centre of the study is the most critical limiting factor. More research on SAGIT is undoubtedly needed (especially on large groups of subjects) to explore the positive aspects of this tool and also to learn about its limitations. Nevertheless, the SAGIT tool turns out to be valuable and reliable in the assessment of disease activity. It might be helpful in the therapeutic decision-making process in patients with acromegaly.

## Conclusions

The SAGIT instrument is easy to use even when completed in the retrospective medical record review. Our study indicates its potential utility for distinguishing clinical stages of acromegaly in patients from Poland. Therefore, we recommend that SAGIT be included in the patient's medical records.

## Data Availability

The datasets used and/or analysed during the current study are available from the corresponding author on reasonable request.

## Abbreviations

GH: Growth Hormone
IGF-1: Insulin-like Growth factor
MRI: Magnetic resonance imaging
CT: Computed tomography
IQR: Interquartile range
BMI: Body mass index
OGTT: Oral glucose tolerance test

## References

[1] Melmed, S. Acromegaly pathogenesis and treatment. *J. Clin. Invest*. **119**(11), 3189–3202 (2009). https://www.mp.pl/paim/issue/article/16232. 10.1172/JCI39375 PMC2769196 19884662

[2] Lugo G, Pena L, Cordido F (2012). Clinical manifestations and diagnosis of acromegaly. Int. J. Endocrinol..

[3] Vilar L, Vilar CF, Lyra R, Lyra R, Naves LA (2017). Acromegaly: Clinical features at diagnosis. Pituitary.

[4] Webb SM (2006). Quality of life in acromegaly. Neuroendocrinology.

[5] Solomon E, Brănișteanu D, Dumbravă A, Solomon RG, Kiss L, Glod M (2019). Executive functioning and quality of life in acromegaly. Psychol. Res. Behav. Manag..

[6] Abreu A, Tovar AP, Castellanos R, Valenzuela A, Giraldo CMG, Pinedo AC (2016). Challenges in the diagnosis and management of acromegaly: A focus on comorbidities. Pituitary.

[7] Powlson AS, Gurnell M (2016). Cardiovascular disease and sleep-disordered breathing in acromegaly. Neuroendocrinology.

[8] Hannon AM, Thompson CJ, Sherlock M (2017). Diabetes in patients with acromegaly. Curr. Diab. Rep..

[9] dos Santos MCC, Nascimento GC, Nascimento AGC, Carvalho VC, Lopes MHC, Montenegro R (2013). Thyroid cancer in patients with acromegaly: A case-control study. Pituitary.

[10] Dworakowska D, Grossman AB (2019). Colonic cancer and acromegaly. Front. Endocrinol. [Internet]..

[11] Gatto F, Campana C, Cocchiara F, Corica G, Albertelli M, Boschetti M (2019). Current perspectives on the impact of clinical disease and biochemical control on comorbidities and quality of life in acromegaly. Rev. Endocr. Metab. Disord..

[12] Wen-Ko C, Szu-Tah C, Feng-Hsuan L, Chen-Nen C, Ming-Hsu W, Jen-Der L (2016). The impact of diabetes mellitus on the survival of patients with acromegaly. Endokrynol. Pol..

[13] Melmed S, Bronstein MD, Chanson P, Klibanski A, Casanueva FF, Wass JAH (2018). A Consensus Statement on acromegaly therapeutic outcomes. Nat. Rev. Endocrinol..

[14] Bernabeu I, Aller J, Álvarez-Escolá C, Fajardo-Montañana C, Gálvez-Moreno Á, Guillín-Amarelle C (2018). Criteria for diagnosis and postoperative control of acromegaly, and screening and management of its comorbidities: Expert consensus. Endocrinol. Diabetes Nutr..

[15] Wolters TLC, Roerink SHPP, Sterenborg RBTM, Wagenmakers MAEM, Husson O, Smit JWA (2020). The effect of treatment on quality of life in patients with acromegaly: A prospective study. Eur. J. Endocrinol..

[16] Giustina A, Bevan JS, Bronstein MD, Casanueva FF, Chanson P, Petersenn S (2016). SAGIT: Clinician-reported outcome instrument for managing acromegaly in clinical practice–development and results from a pilot study. Pituitary.

[17] Giustina A, Bronstein MD, Chanson P, Petersenn S, Casanueva FF, Sert C (2019). Staging and managing patients with acromegaly in clinical practice: Baseline data from the SAGIT validation study. Pituitary.

[18] Giustina A, Bronstein MD, Chanson P, Petersenn S, Casanueva FF, Sert C (2021). International multicenter validation study of the SAGIT instrument in acromegaly. J. Clin. Endocrinol. Metab..

[19] Varlamov EV, Niculescu DA, Banskota S, Galoiu SA, Poiana C, Fleseriu M (2021). Clinical features and complications of acromegaly at diagnosis are not all the same: Data from two large referral centers. Endocr. Connect..

[20] Katznelson L, Laws ER, Melmed S, Molitch ME, Murad MH, Utz A (2014). Acromegaly: An endocrine society clinical practice guideline. J. Clin. Endocrinol. Metab..

[21] Publication ethics of human studies in the light of the Declaration of Helsinki—a mini-review. J. Med. Sci. [Internet] (accessed 15 Jul 2022); https://jms.ump.edu.pl/index.php/JMS/article/view/700.

[22] Vila G, Jørgensen JOL, Luger A, Stalla GK (2019). Insulin resistance in patients with acromegaly. Front. Endocrinol..

[23] Ferraù F, Albani A, Ciresi A, Giordano C, Cannavò S (2018). Diabetes secondary to acromegaly: Physiopathology, clinical features and effects of treatment. Front. Endocrinol..

[24] Bolanowski M, Adnan Z, Doknic M, Guk M, Hána V, Ilovayskaya I (2022). Acromegaly: Clinical care in Central and Eastern Europe, Israel, and Kazakhstan. Front. Endocrinol. [Internet]..

[25] Halse J, Haugen HN (1980). Calcium and phosphate metabolism in acromegaly. Acta Endocrinol. (Copenh)..

[26] Xie T, Tian P, Wu S, Zhang X, Liu T, Gu Y (2020). Serum phosphate: Does it more closely reflect the true state of acromegaly?. J. Clin. Neurosci. Off. J. Neurosurg. Soc. Austral..

[27] Constantin T, Tangpricha V, Shah R, Oyesiku NM, Ioachimescu OC, Ritchie J (2017). Calcium and bone turnover markers in acromegaly: A prospective, controlled study. J. Clin. Endocrinol. Metab..

[28] Manroa P, Kannan S, Hatipoglu B, Licata A (2014). Hypercalcemia and acromegaly-clarifying the connections: A case report and review of the literature. Endocr. Pract..

[29] Shah R, Licata A, Oyesiku NM, Ioachimescu AG (2012). Acromegaly as a cause of 1,25-dihydroxyvitamin D-dependent hypercalcemia: Case reports and review of the literature. Pituitary.

[30] Shi S, Zhang L, Yu Y, Wang C, Li J (2021). Acromegaly and non-parathyroid hormone-dependent hypercalcemia: A case report and literature review. BMC Endocr. Disord..

[31] Halupczok AJ, Jawiarczyk-Przyby A, Bolanowski M (2015). Patients with active acromegaly are at high risk of 25(OH)D deficiency. Front. Endocrinol. [Internet]..

[32] Colao A, Amato G, Pedroncelli AM, Baldelli R, Grottoli S, Gasco V (2002). Gender- and age-related differences in the endocrine parameters of acromegaly. J. Endocrinol. Invest..

[33] Park SH, Ku CR, Moon JH, Kim EH, Kim SH, Lee EJ (2018). Age- and sex-specific differences as predictors of surgical remission among patients with acromegaly. J. Clin. Endocrinol. Metab..

[34] Bogusławska, A. *et al*. Gender and age differences among patients with acromegaly. *Pol. Arch. Intern. Med.* (2022; accessed 21 May 2022); https://www.mp.pl/paim/issue/article/16232. 10.20452/pamw.16232 35289160

